# Aerobiology: Experimental Considerations, Observations, and Future Tools

**DOI:** 10.1128/AEM.00809-17

**Published:** 2017-08-17

**Authors:** Allen E. Haddrell, Richard J. Thomas

**Affiliations:** a School of Chemistry, University of Bristol, Bristol, United Kingdom; b Defence Science and Technology Laboratory, Porton Down, Salisbury, Wiltshire, United Kingdom; Rutgers, The State University of New Jersey

**Keywords:** aerosol survival, atmospheric transport, bioaerosol

## Abstract

Understanding airborne survival and decay of microorganisms is important for a range of public health and biodefense applications, including epidemiological and risk analysis modeling. Techniques for experimental aerosol generation, retention in the aerosol phase, and sampling require careful consideration and understanding so that they are representative of the conditions the bioaerosol would experience in the environment. This review explores the current understanding of atmospheric transport in relation to advances and limitations of aerosol generation, maintenance in the aerosol phase, and sampling techniques. Potential tools for the future are examined at the interface between atmospheric chemistry, aerosol physics, and molecular microbiology where the heterogeneity and variability of aerosols can be explored at the single-droplet and single-microorganism levels within a bioaerosol. The review highlights the importance of method comparison and validation in bioaerosol research and the benefits that the application of novel techniques could bring to increasing the understanding of aerobiological phenomena in diverse research fields, particularly during the progression of atmospheric transport, where complex interdependent physicochemical and biological processes occur within bioaerosol particles.

## INTRODUCTION

Aerosols injected into the atmosphere from the biosphere (bioaerosols) account for a significant portion of all atmospheric aerosols (1). Despite their low numbers relative to other natural aerosols, bioaerosols (whose sources include microorganisms contained within wind-blown dust and sea spray) are speculated to impact climate through their behavior as efficient cloud condensation nuclei (2, 3). Biological aerosols are also important from the perspective of human health, as they are intimately involved in the transmission of many respiratory pathogens (4, 5).

Risk analysis modeling aims to develop predictive models of transmission and infection based on laboratory generation of aerosols containing respiratory pathogens. These experimental models are invaluable for understanding epidemic transmission, developing infection control measures, and advising bioterror preparedness for public health (6–8). Effective risk modeling requires an in-depth understanding of experimental aerosol techniques and their potential impact on the final outcome, whether that is aerosol decay, transmission rate, or infectious dose.

This article reviews the current understanding, advances, and limitations in laboratory aerobiological studies, where the relationship between microorganism preparation, aerosol generation, evaporation, transport, and fate cumulatively may affect the final outcome of inhalational infection or survival in the environment. In this review, the term “bioaerosol” is limited explicitly to infectious aerosol droplets containing living species, specifically bacteria and viruses; the study of this subset of bioaerosols comes with its own unique set of challenges that need to be recognized and addressed. The PubMed database was searched to identify relevant studies using the following strings: aerosol AND survival, bioaerosol AND generation, bioaerosol AND sampling. The terms bacteria and virus were interchanged for the term survival in the first search string; only published studies were included. References with no relation to bioaerosol, defined as “infectious aerosol droplets” (e.g., fungal spores, pollen) were generally discarded unless the technology could be applied to the field. Retrieved studies were also reviewed for additional references. Although intrinsically linked to the general theme of this review, the development of inhalational animal models to replicate human disease is considered outside the scope of this review, and readers are directed to the extensive literature in this field (e.g., 9–11).

## AEROSOL GENERATION, SAMPLING, AND POSTPROCESSING CONSIDERATIONS

Aerosol generation and sampling prior to microbiological analysis are conducted for a range of bioaerosol-related research activities (e.g., determination of aerosol decay rates and inhalational infectious dose, efficacy of decontamination strategies, and evaluation of bioaerosol sampling technologies). These dynamic processes can cause damage due to shear forces acting on the microbial cells (12–27). Table 1 outlines some major aerosol generators and samplers used in aerobiological studies and their operating mechanisms. The majority of studies use reflux aerosol generators in conjunction with impingement to collect the generated aerosol. This system can be safely used in biocontainment laboratories for inhalational challenges and aerosol fate studies. However, comparative studies show that refluxing nebulizers produce the greatest loss of physiological function as a function of time in bacteria (16, 19–21, 24). The loss of function has been linked to membrane damage (13, 20, 24), release of ions into the medium (e.g., PO_4_^2−^) (28), cell fragmentation (15, 23), reduction in ATP activity (27), and magnitude of associated electrical charge (29), as the bacteria remaining in the nebulizer repeatedly pass through the device nozzles. Similar effects are observed for viruses (25). Repair of bacterial cells damaged by nebulization appears to be an energy-dependent process with a requirement for divalent cations, although independent of *de novo* RNA or protein synthesis (13, 30); it is unlikely that repair occurs in viruses due to their reliance on host cell factors for protein transcription and translation. In contrast, it has been reported that damage is reduced in nonrefluxing aerosol generators, in which the microorganisms pass through the nozzle once (16, 24).

**TABLE 1:** Methods used to generate and sample microbial aerosols useful for aerosol fate and inhalational infection research^*a*^

Step in generation and mechanism	Apparatus example(s)	Description	Reference(s)
Aerosol generation			
Reflux nebulization (1-, 3-, and 6-jet versions commonly used)	Collison nebulizer, Wells atomizer, TSI 9302, FK-8 aerosol gun, Aeroneb Lab	Refluxing two-fluid atomizer operates via Venturi effect and wall impaction; liquid recirculation occurs every 6 s in the 3-jet version (134)	14, 16, 20, 23–25, 78, 79, 98, 121, 161–166
		Increased jet numbers increase the rate of aerosol generation and recirculation; reservoir evaporation occurs over time, causing concn effects	
		Generally used for liquids, although the Wells atomizer was used for dry powders; particle sizes are small, 0.7–2.2 μm	
		Forces associated with reflux nebulization can cause deagglomeration of aggregates, leading to observed increase in bacterial concn in spray suspension	
Nonreflux nebulization	Single-pass aerosolizer	Atomization (as described above) without wall impaction and recirculation	24
Aerosol bubbling	SLAG^*b*^ and variants	Liquid dripped onto a membrane is broken into droplets by airflow through the membrane	16, 24, 26
		Droplets burst due to increased pressure gradient inside vs outside the device, generating small aerosol particles	
Centrifugal atomization	Spinning top aerosol generator	Centrifugal forces move liquid applied to rotating disc toward edges, producing ligands that break into droplets	167
Flow focusing	FFAG,^*c*^ C-Flow nebulizer	Liquid flows through an orifice forming microjets that break up into particles by aerodynamic suction of an accelerated air stream	20, 24, 168
		Good monodispersity of droplets can be achieved	
Aerosol sampling			
Impingement	Impingers^*d*^ (AGI-4, AGI-30, AGI model 7541 AGI); SKC biosampler	The aerosol accelerates through a critical orifice, causing inertial impaction into liquid	17, 18, 21, 22, 169–177
		Efficiency is affected by physical parameters (e.g., sampling flow rate, nozzle no. and angle, distance of nozzle from liquid, solution type and vol, particle bounce, prolonged sampling time [liquid evaporation, increased damage], and binding of microorganisms to collection vessel wall)	
		Reaerosolization can occur due to liquid bubbling	
		Addition of glass beads can increase virus collection efficiency	
		SKC biosampler possesses three angled nozzles, creating gentler swirling motion of bioaerosol during collection	
		AGI-30 impaction velocity reaches 265 m/s (the velocity is much reduced in other samplers)	
Impaction	Single or multistage impactors: Andersen, Mercer, Ultimate, MAS-100, Burkard	Operate at constant flow rates, with air flowing through orifice causing inertial impaction of particles too large to remain entrained in airflow; size fractionation possible	21, 22, 46, 178–180
		Collection onto a range of different substrates (e.g., agar plates, gelatin-coated slides, filters) possible	
		Substrate choice can affect collection efficiency due to effects on microbial viability and particle bounce	
		In the Burkard and sixth stage of Andersen impactors, impaction velocities reach 12 and 24 m/s, respectively	
Filtration and impaction	Gelatin filter, nitrocellulose, polycarbonate	Greater physical sampling efficiencies; biological sampling efficiency may be lower due to sensitivity of collected microorganisms to air drawn past filter	21, 22, 47, 48
		Elution of material from filter surface (e.g., vortexing, shaking, solution vol and type) can influence efficiency	
Direct capture	Microthreads	Particles collected onto fine microthreads (e.g., spider silk, glue thread) are wound onto a frame	77, 95–97, 122–124
Cyclonic separation	NIOSH cyclonic biosampler	Airflow drawn into cylindrical container that is rotated, causing larger particles to deposit and collect on walls by centrifugal force	25, 34
Electrostatic precipitation	Ionizers (AS150, model 3100 aerosol sampler)	Airborne particles are electrically charged and subjected to electric field, causing gentle deposition velocity onto collection substrate	29, 35, 181
		Bioefficiency for spores is greater than for Gram-negative bacteria	
		Impaction velocities reach 0.01–1 m/s	
Animal inhalation	Rodents, primates	Aerosol particles regionally deposited due to inertial impaction, sedimentation, diffusion, interception, and electrostatic effects in respiratory tract	182
		Deposition is a function of airway geometry and particle properties (e.g., size, shape, density, hygroscopicity)	

a Note that the list is merely representative and not exhaustive. Researchers are recommended to conduct rigorous validation of the aerosol experimental system for each individual microorganism tested.

b Sparging liquid aerosol generator.

c FFAG, flow-focusing aerosol generator.

d All-glass impinger.

Sampling methods for airborne microorganisms include impingement, impaction, filtration, cyclonic separation, and electrostatic precipitation. This review will not cover all bioaerosol samplers; rather, we selected the main sampling mechanisms and representative sampler models. The reader is directed to a couple of comprehensive reviews on bioaerosol sampling for further details (31, 32). Each sampling technique has advantages and disadvantages for sampling microbial aerosols (Table 1) with the potential to cause microbial damage. Dependent on the microbe, this damage may be transient; for example, impingement (AGI-30; 15 to 60 min) causes structural damage to Pseudomonas fluorescens cells with recovery achieved on nonselective media (15). Aerosol sampling times for determining the infectious dose and aerosol decay rates generally range from 1 to 10 min, a period which minimizes the effects of microbial damage (22, 33). However, for infectious aerosols there are few comparative studies of the bioefficiencies of different sampling mechanisms. Where studies comparing samplers have been conducted, differences between microbial structures influenced sampler bioefficiency; for example, infectivity and culturability differences were observed between bacteriophages and influenza A virions sampled by the SKC biosampler and NIOSH cyclone (25, 34). Similar species-dependent effects have been observed for bacteria in terms of sampling bioefficiency; in particular, Bacillus spp. endospores tend to be less affected by aerosol sampling method (15, 17, 21, 22). One reason for differences in sampler bioefficiency is variations in sampling velocities; for impingement, the velocity reaches 260 m/s, 10-fold greater than other samplers (35) (Table 1). Second, the rapid rehydration that occurs during sampling can be detrimental to microorganisms (36–38).

Minimizing stresses that occur during aerosol generation and sampling is hence critical to accurate representation of aerosol decay and infectivity. Aerosol generation stresses can be reduced by using single-pass devices that reduce the probability of microorganisms being damaged (24). Depending on sampler choice, maximizing recovery of microbes can be achieved in a number of ways. Prolonged sampling times are a consistent cause of reduced viability, and hence collection times across all types of samplers and should be minimized (22, 39). The cell membrane is a major site of damage for Gram-negative bacteria when aerosolized as sampled, as demonstrated by increased sensitivity to hydrolytic enzymes (12). Impingement requires collection into a liquid which can be optimized to reduce osmotic shock and maximize repair and recovery. For example, addition of compatible solutes and scavenging enzymes (e.g., trehalose, raffinose, polyhydric alcohols, betaine, and catalase) can facilitate survival following the stresses associated with aerosol generation, transport, and sampling (37, 40–45). Particle bounce and viability loss in impactors for vegetative Bacillus subtilis and Escherichia coli cells were reduced by applying a thin film of mineral oil, which significantly enhanced collection efficiency (46). Filtration methods provide high physical collection efficiencies, but bioefficiency can be dependent on filtration time and postprocessing procedures (21, 24, 47, 48). A major problem with filtration samplers is continued drawing of air through the filter desiccate of collected microorganisms in a time-dependent manner. However, filtration onto gelatin membranes provides a medium that retains moisture and can be placed into warm media to recover collected microorganisms providing good bioefficiency (21, 24).

Postsampling enumeration and storage are additional considerations. Enumeration can introduce error, as organisms can be sensitive to impaction onto an agar surface (49), sensitive to the plating medium (15), and sensitive to the process of spread plating (50–52). Direct methods, such as microscopy or flow cytometry, in conjunction with various dyes or quantitative PCR can indicate physiological activity of the collected microorganisms (15, 17, 53). Storage temperature, sampling solution, and length of time can prompt microbial replication (or death), causing misrepresentation of the actual viability of the sampled bioaerosol (47). Samples should be processed as soon as possible after aerosol sampling; however, this is highly dependent on the microorganism. For example, Bacillus spp. endospores have been demonstrated to be less affected by storage temperature (4 and 25°C) than Escherichia coli; however, compared to immediate enumeration, both species had increased counts after extended periods of storage at 25°C (10 and 24 h for B. subtilis and E. coli, respectively), indicating significant disaggregation and/or multiplication in the collection medium, which in this case was sterile deionized water containing a small quantity of detergent (47).

The data indicate that the method of aerosol generation can damage the microorganism at the subcellular level, at the very least subtly, and influence resultant estimates of microbial viability in the aerosol phase. None of these mechanisms is entirely representative of the natural transmission mechanisms of respiratory pathogens, e.g., coughing and sneezing followed by deposition in the respiratory tract (4, 5). The complexity of fluid fragmentation and droplet formation of oro-respiratory secretions during coughs and sneezes has recently been elucidated, with the viscoelastic properties of respiratory secretions playing a defining role in final droplet size (54, 55). Viscoelasticity of respiratory secretions will change with anatomical location (e.g., nasal, bronchial) and disease state (e.g., chronic bronchitis, sinusitis, cystic fibrosis) as a result of changes in mucin content, which will also affect droplet sizes (56, 57). Natural aerosol transmission events are likely to be less violent than the aforementioned aerosol generation processes. Therefore, selection and validation of experimental regimens (aerosol generator, spray fluid composition, and sampling) to minimize microbial damage, promote maximal recovery, and most closely replicate the natural event being modeled are important for interpretation of aerosol data used in risk analysis models. Based on this review and also more extensive reviews on sampling methodology (31, 32), it is apparent that given the variability in microorganism responses to the stresses of aerosol generation and collection, it is advisable to perform method validation for each particular microorganism. Testing a range of aerosol generators and samplers to ensure that the behavior of the microorganism within the system is understood facilitates appropriate selection of apparatus and methodology to maximize recovery during enumeration.

## AEROSOL TRANSPORT AND PHYSICAL PROCESSING

The physicochemical properties of bioaerosol particles govern all of the biological processes within. The conditions in a bioaerosol particle that a microorganism will experience can be dramatically different than those in bulk liquid; the solute concentrations commonly reach supersaturation (58), while the rate of water transport within the droplet can vary by orders of magnitude (59). Both of these properties are regulated by the total water present in the droplet. Thus, a detailed understanding of the hygroscopic properties of a bioaerosol as a function of solute composition (including biological species itself) is critical for understanding and predicting longevity and overall infectivity.

The typical trajectory in relative humidity (RH) for a respiratory pathogen would be from a high level at the point of dispersion (>95%), to a low level during atmospheric transport (ambient RH), to a high level upon inhalation (>95%) (60). During its lifetime, the water activity (a_w_) within a droplet equilibrates with the atmospheric RH through either the addition or removal of water (61). From droplets larger than 100 nm in size, the water activity is equal to the gas-phase RH at equilibrium. The rate at which this mass flux occurs and the final particle size attained are a reflection of the temperature and humidity of the gas phase of the aerosol and the droplet solute (62, 63). Importantly, all microorganisms require water for activity of critical enzyme-driven biochemical reactions (e.g., respiration). Interestingly, in studies looking at osmotic tolerance in bulk liquid phase, depending on the bacterial species, multiplication and growth are inhibited at a_w_ values of 0.86 to 0.97, with further reductions inducing dormancy or eventually reducing viability (64, 65).

The hygroscopic behavior of any multicomponent aerosol is dependent on the relative abundance of each chemical species in the solute, where each component will contribute a proportion to the uptake or loss of water (61). This paradigm holds true for bioaerosols; for example, it has been shown that the solute concentration affects hygroscopic growth of aerosolized B. subtilis and Pseudomonas fluorescens vegetative cells (66). However, to study the hygroscopic behavior of an aerosol where the aim is to generate predictive models, much information about the solute is required. The relative abundance of each component within the aerosol is mandatory (67–71), as is a detailed understanding of how the various components within the solute interact with one another (72). While this is somewhat straightforward with regard to nonbiological aerosols, it remains a major challenge with bioaerosols. For example, infected individuals coughing and sneezing will produce larger droplets with different concentrations of mucus and other organic and inorganic solutes than those produced by healthy individuals (57). Similarly, in laboratory studies, microbial culture conditions (liquid broth, solid agar, and nutrient composition) and growth phase affect the concentration and types of nutrients present in the spray suspension, and these factors influence aerosol survival (25, 73–77). Indeed, survival of a viral simulant, the bacteriophage MS2, differed in human-derived saliva, artificial saliva, and cell culture medium, with the greatest decay observed in human-derived saliva (78). This has been observed for other viruses and bacteria upon comparing survival after aerosolization from body fluids (natural or synthetic) and culture medium (79–82). This highlights the caution needed in extrapolation of results from experimental to *in vivo* situations being modeled in a risk analysis.

The primary challenge in experimental studies of the factors that regulate the hygroscopic behavior of a bioaerosol is to control and know the complete composition of the bioaerosol droplets. For example, a simple factor such as control of the number of organisms per droplet/particle is not trivial when using conventional aerosolization processes. To attempt to address this specific issue in studies of laboratory-generated bioaerosols, a particular size is selected for a nebulized and dried bioaerosol sample, allowing estimation of the number of species per droplet prior to hygroscopic analysis (16). For more complex (and atmospherically relevant) bioaerosols, the hygroscopic behavior of an anthropogenic bioaerosol has been estimated indirectly (83, 84). In these studies, the relative growth in bioaerosol particle size with increases in RH was estimated through correlation analysis between the temporal size distributions (aerodynamic diameter) of airborne fungi with meteorological information (RH).

Thermodynamic models to predict the hygroscopic behavior of aerosol (e.g., universal quasichemical functional group activity coefficients [UNIFAC]) have been used for bioaerosols to limited success (58, 85). Generally, these models are able to predict the hygroscopic behavior of large and complex organic molecules through parameterization of the functional groups present (such as carboxylic acids) (86). Even though, organically, bioaerosol consists primarily of sugar alcohols and highly polar sugars (87), it remains unclear the extent to which these models can be used to predict the hygroscopic behavior of bioaerosols (88). The reason for this is that even when the relative abundances of functional groups and chemical species within a single bioaerosol droplet are known, the accumulation of noncovalent interactions between these species is not. The presence of cellular membranes within the droplet could kinetically limit the hygroscopic behavior of all the chemical species within the aerosol.

The limited number of comprehensive studies that have explicitly focused on the physicochemical properties of bioaerosols is problematic. Their absence has constrained the means by which the longevity of a suspended bioaerosol can be investigated.

## DETERMINING BIOAEROSOL LONGEVITY

Bioaerosol longevity is simply the length of time in which a biological species will remain either infectious or viable while suspended as a single particle. In an ideal experiment, the entire composition of the target bioaerosols would be explored; as discussed in previous sections, this is technically challenging due to the selectivity of samplers and the heterogeneity of bioaerosol composition. Despite this, numerous studies on bioaerosol longevity have been published.

Techniques for investigating survival of bioaerosols *in vitro* (Table 2) maintain the particles either in the air column (i.e., “dynamic bioaerosols”) or captured on a fine substrate such as spider silk or glue fibers (i.e., “captured bioaerosols”). The rotating drum is probably the standard procedure used for aerosol longevity studies, based on the seminal design of Goldberg and colleagues (89). Modifications have permitted greater control (e.g., *in situ* monitoring of parameters) and accessibility to a range of environmental parameters (e.g., temperature, UV, volatile organic compounds) and the suspension of larger aerosol particle sizes for sufficiently long periods of time (90–93). Methods based on capturing bioaerosols on microfibers derived from spider escape silk and glue gun fibers have been utilized with success (77, 94–96). Comparative studies on filoviruses have demonstrated that microthread-captured bioaerosols decay at a similar rate as those held dynamically within rotating vessels (33, 97).

**TABLE 2:** Examples of experimental techniques used to study the fate of microorganisms in aerosols

Device	Mechanism	Aerosol state^*a*^	Outdoor use?	Reference(s)
Rotating drum	Rotational speed of drum prevents aerosol from settling for period of time dependent on particle size	Dynamic	N	33, 81, 82, 92, 93, 98, 110, 125, 162, 183
Microthread	Aerosol captured on spider microthreads or glue fibers wound around a metal frame that can be slotted into an exposure apparatus	Captured	Y	77, 95–97, 122–124, 129
Sphere	Steel sphere with mixing fans	Dynamic	N	123, 184
Aerosol chamber	Large chambers with mixing fans	Dynamic	N	185
Greenhouse	No mixing fan	Dynamic	Y	186, 187

a Dynamic refers to particles maintained as a buoyant aerosol, while captured refers to aerosol particles immobilized on a substrate.

The methods for retention of microorganisms in the aerosol phase have been used extensively to determine biological decay in the airborne state as a function of time and under a range of environmental conditions (Table 3). The aerosol is sampled at time intervals and the number of viable microorganisms is determined, enabling calculation of the aerosol decay rate. Sampling method and subsequent microbiological processing and enumeration can alter the number of recovered microorganisms (15, 17, 21, 22). Therefore, it is important to minimize microbial stress during aerosol collection to facilitate accurate calculation of the decay rate. During method validation, it is important to differentiate biological decay from physical losses due to deposition on the walls of the vessel or removal from the microthreads due to turbulence (or the presence of antimicrobial substances on the silk). Physical loss in aerosol systems is determined by using physical tracers that will not biologically decay, such as Bacillus spores, chemicals (e.g., fluorescein), or polymer beads (21, 98, 99). The decay rates of the target microorganism and the physical tracer can be compared and the true biological decay rate determined.

**TABLE 3:** Atmospheric, environmental, and microbial factors that affect survival and infectivity of airborne microorganisms

Factor	Description	References^*a*^
Relative humidity	Levels studied generally from 20 to 90% RH	40, 44, 75, 79, 81, 98, 99, 112, 114, 162, 183, 188–192
Temperature	Wide ranges studied, from subzero to 50°C	79, 163, 190, 191, 193
Solar radiation	Variability in spectra examined but inclusive of UV-A and UV-B wavelengths	45, 77, 114–117, 187
Oxygen	Generation of ROS^*b*^ during aerosol transport	43, 104–108, 164, 194
Ozone	Reactive with pollutant gases and pinenes	121, 185
Pollutant gases, OAF	CO, SO_2_, NO_2_, ethene, cyclohexene, and SOAs (e.g., alkenes, turpenes)^*c*^	30, 92, 121–130, 184
Wet/dry prepn	Droplets or dried particles	75, 111, 162, 188, 195
Growth phase	Exponential or stationary	30, 164
Particle size	Microbial aggregates have greater survival than single microorganisms	30, 77, 129, 194
Aerosol age	Infectivity decreased prior to culturability with extended time in aerosol	196–198

a The list of relevant references is reflective and not exhaustive.

b ROS, reactive oxygen species.

c SOAs, secondary organic aerosols. Turpenes are volatile cyclic unsaturated hydrocarbon molecules released by plants.

A disadvantage of these techniques is that they sample bulk aerosols, and it is difficult to develop an appreciation of microenvironment heterogeneity occurring within individual aerosol droplets from the physicochemical and biological perspectives. For example, each individual aerosol droplet is likely to have a different chemical composition, exacerbated by differences in particle size that manifest themselves biologically on the microorganisms incorporated within the droplets. Such differences may be a source of variability in how microbes respond and survive aerosol transport.

## ENVIRONMENTAL FACTORS AFFECTING MICROBIAL LONGEVITY DURING ATMOSPHERIC TRANSPORT AND BACTERIAL SURVIVAL MECHANISMS

A large number of environmental and meteorological factors can influence microbial survival during aerosol transport (Table 3), and to provide greater context for interpretation of results the environmental features of the sampling site should be described. The fate of the microorganism is likely dictated by its physiological status, which is a combinatorial consequence of the atomization process (e.g., spray device, cough, sneeze) with the associated evaporative stresses of aerosol transport and rehydration during inhalation (or sampling into liquid). The mechanisms by which the microorganisms perish have been partially elucidated and depend on the composition of the droplet and surrounding atmosphere.

Atmospheric oxidants (e.g., reactive oxygen and nitrogen species, sulfur dioxide, ozone) will impact microbial longevity by acting either directly on the organism or with constituents within the aerosol droplet (100, 101). The presence of oxygen has been demonstrated to have a deleterious effect on airborne coliform bacteria, particularly at RH less than 40%, and is hypothesized to be due to production of reactive oxygen species by Maillard reactions (30, 102). Maillard reactions are amino-carbonyl reactions that occur between amino groups on proteins and reducing sugars that cause oxidation of macromolecules and death in microorganisms (103). In airborne microorganisms, these reactions may be the cause of oxidative damage to critical enzymes (43, 104–106), phospholipids, and nucleic acids, causing at the molecular and physiological levels of the bacterial cell (i) metabolic imbalance, (ii) membrane destabilization, and (iii) reduction of repair activity (30). Interestingly, recently Maillard chemistry has been implicated as a source of organic compounds within atmospheric aerosols altering particle viscosity and hence the diffusivity rate of water and reactive gases (107). Bioaerosols (including virus, vegetative bacteria, spores, and peptides) subjected to atmospheric ozone concentrations and variations in RH showed temporal changes in fluorescence spectra related to oxidation and hydrolysis of tryptophan (108–110). Although survival is generally greater at higher RH (>80%), certain values (i.e., 70 to 85% RH for E. coli B) (40, 43) produce a large decrease in aerosol survival (40, 106, 111, 112). Likewise, RH-dependent changes in salt concentrations and pH within droplets influence virus viability causing conformational changes in surface proteins and membrane fluidity affecting infectivity (113).

Solar irradiation and atmospheric pollutant gases (including open air factor [OAF]) are two further environmental parameters that can significantly affect longevity in the aerosol phase. Solar irradiation markedly decreased viability compared to control conditions that simulated the night (45, 77, 114–117). Particle size-dependent survival from solar irradiation has been observed, with bacterial clusters persisting for longer periods (77, 116). Terrestrial solar spectral irradiance varies through the day, with season, and with geographical location (118). The UV wavelengths are of most importance for inactivating microorganisms (115, 116), where UV-A and UV-B reach the troposphere with the potential to cause a variety of DNA genomic lesions and damage to nucleic acids, proteins, and lipids due to generation of reactive oxygen species (119, 120). It is important that studies using both simulated and natural solar irradiation report variables such as solar intensity as accurately as is reasonably possible to facilitate data interpretation and standardization between laboratories.

Atmospheric constituents, such as various pollutant gases and secondary organic aerosols (SOAs) (Table 3) have been demonstrated to have significant deleterious effects on aerosol longevity (30, 92, 121–129). Many of these may contribute to a phenomenon known as open air factor, where aerosolized microorganisms exposed to open climatic conditions decay more rapidly than those in enclosed laboratory vessels subjected to similar temperature and RH (30, 122–124, 128, 129). The precise nature of OAF is not fully understood but is hypothesized to involve a number of highly reactive products (e.g., hydroxyl radicals) from photochemical interactions between ozone and unsaturated hydrocarbons from anthropogenic sources (e.g., engine-related alkenes) and nonanthropogenic sources (e.g., plant turpenes) (30, 122). The reactive species rapidly oxidize and degrade macromolecules such as lipids, proteins, and nucleic acids (30, 130). The effect of OAF is enhanced at high humidity (80 to 90% RH) for both E. coli and Micrococcus albus (122). Such humidity effects warrant further investigation in relating the increased water content of aerosol particles at higher humidity.

How microbes regulate and survive aerosol transport is undetermined. Evidence suggests that the ability for transcription and translation to occur in the environment of an evaporating droplet is reduced (30, 131, 132). Evaporation and rehydration of aerosol particles imparts osmotic and desiccative stresses on the microbe that are reflective of the humidity of the surrounding atmosphere and composition of the particle. The molecular response of many bacterial species to osmotic stress and desiccation is well documented from research understanding survival in food matrices, aquatic and marine systems, and terrestrial environments (65). Hyperosmotic stress (i.e., reduced a_w_) causes a reduction in cytoplasmic volume as water exits the bacterium; concomitantly, cell growth and respiration cease as the bacterium adapts to the hyperosmotic conditions. Initially charged solutes (e.g., K^+^ ions, glutamate) are accumulated via specific uptake mechanisms (65, 133–135). Interestingly, the inability to control efflux of K^+^ ions is correlated with decreased survival in aerosolized E. coli cells (28, 136). Synthesis of compatible solutes (e.g., trehalose) or uptake from the surrounding medium (e.g., glycine betaine, proline) stabilizes proteins, enzymes, and membrane phospholipids to enable critical biochemical processes to continue in hyperosmotically stressed bacteria. As the bacterial cell stabilizes, a number of proteins are synthesized, prompting repair of DNA damage, scavenging of reactive oxygen species, and degradation of misfolded proteins (65, 133–135). Osmotically adapted cells often show cross-tolerance to other stresses, such as high temperature and oxidative shock (137). Recently, E. coli subjected to a rapid downshift in a_w_ (0.993 to 0.960) in medium was demonstrated to control protein misfolding by transient expression of the RpoE and RpoH regulons in conjunction with the RpoS regulon to facilitate prolonged adaptation to the hyperosmotic conditions (138).

The molecular studies described above have all been conducted in bulk solution phase and expose the microorganisms to hyperosmotic stress. Microorganisms will be exposed to hyperosmotic conditions within an evaporating droplet (i.e., low a_w_ conditions), enabling speculation that similar molecular mechanisms play role in bacterial survival within evaporating aerosol droplets. As discussed below, advances in atmospheric chemistry and single-cell genomic techniques will allow investigation of whether similar molecular mechanisms occur in an aerosol droplet as a function of evaporation rate and droplet composition. Importantly, if airborne microorganisms can induce adaptive responses promoting survival, then there is the potential that colonization and infection of the respiratory tract is primed while the bacteria are transported in the atmosphere. Any induced virulence factors would offer attractive targets for combating respiratory infection.

## NEW TECHNIQUES FOR ADVANCING AEROSOL SCIENCE AND AEROBIOLOGY

Bioaerosols, even when produced under controlled laboratory conditions, are complex. They are generally polydispersed in terms of both physicochemical and biological properties, and the heterogeneity in the nature of a bioaerosol evolves with time and distance from the source. Technological advances in the fields of aerosol science and molecular biology are timely to facilitate multidisciplinary approaches to understand heterogeneity at the single-droplet and single-microorganism levels (including microbial aggregates) and to explore the fundamentals of biological decay and survival in aerosol droplets.

Optical techniques, such as optical tweezers and electrodynamic balances, where single aerosol droplets can be captured and levitated within an electric field for periods of time (seconds to days), have been extensively used in atmospheric chemistry to investigate heterogeneous chemistry, phase separation, hygroscopicity, and ice nucleation activity using analytical techniques, including Raman microspectroscopy (139–144). Utilization of these techniques for biological aerosols has been limited to date. However, optically trapped single biological cells in solution produce characteristic Raman scattering signatures (145–148), and E. coli exposed to 1-butanol resulted in spectroscopic and anisotropic detection of real-time phenotypic changes in fatty acid composition and membrane fluidity (148). Although these studies were conducted in liquid bulk solution rather than aerosol droplets, it exemplifies the power of the technology. Furthermore, such techniques are being used to explore individual aerosol particles containing microorganisms, fungal spores, and pollen (149–151). The electrodynamic balance technique has been used to accurately deposit single particles containing respiratory syncytial virus onto airway epithelial cells enabling the cellular response to infection to be analyzed (152). This technique enables interaction at the air-cell interface with single aerosol particles, a more representative scenario than the air-liquid interface studies commonly conducted for *in vitro* infection studies. It is a technique that seems applicable although currently rarely applied to understanding the heterogeneity of bioaerosols at the single-droplet and microorganism level.

Microbial cells respond to environmental stimuli by regulating gene expression resulting in modulation of the quantities and composition of functional proteins available to combat a particular stressful condition. Transcriptional analysis and insertional mutagenesis have been used to identify bacterial genes regulated in response to stresses associated with aerosol survival, such as desiccation and osmotic pressure (135, 153). Currently, these techniques have not been applied to aerosolized microbial populations; however, it can be hypothesized that similar responses may be expected and warrant exploration. The relative abundance of particular proteins critical to aerosol survival will vary from cell to cell. Exploring this heterogeneity at the single-cell level is complicated due to the relatively low abundance of stress-responsive proteins. However, the last 5 years have seen significant advances in molecular techniques enabling exploration of genomics and proteomics (154–157). Techniques for isolating single cells, such as flow cytometry and microfluidics, can be combined with techniques such as PCR and next-generation sequencing for probing the transcriptional response of single cells (158). Indeed, single-cell genomic techniques have been applied to understanding airborne metagenomes in urban settings (159, 160). Application to aerosolized populations in a laboratory setting would seem straightforward. However, care in experimental design is needed to discriminate the true effects of aerosol transport from the stresses of aerosol generation and sampling.

These emerging technologies have the potential to dramatically impact numerous areas of bioaerosol science. They will lead to improved parameterization of the fundamental properties of bioaerosol, such as the interplay between environmental conditions with species longevity and/or gene expression. These data will lead to better predictions of disease dynamics in areas such as general industrial hygiene, animal husbandry, hospital design, and biosecurity. Furthermore, the data collected from these laboratory-based instruments will inform conventional research of environmental samples.

## CONCLUDING REMARKS

Experimental factors affect the microbiological sample taken forward for quantification of infectious dose or biological decay rate. Therefore, a thorough understanding of the sampling and enumeration process is critical to interpretation of the final data set. Furthermore, no single aerosol generation or sampling method is likely to suit all purposes (i.e., size selectivity, species sensitivity); therefore, the experimental apparatus should be selected based on the hypothesis and microorganism being tested and the data interpreted alongside the caveats associated with the methodology. For experiments designed to generate data for input into risk analysis determination of human inhalational exposure then it is recommended that aerosol generators, samplers (and collection fluid) be used that cause minimal damage or promote maximal recovery of the microorganisms during collection to prevent underestimation of risk estimates.

Fundamental questions remain regarding aerosol transmission of respiratory pathogens, particularly the underlying mechanisms of survival and/or death during aerosol transport and the role the microenvironment of the droplet plays as it evaporates then rehydrates during inhalation. However, as outlined in this review, advances in distinct scientific fields could support a systematic dissection of the biological response of microorganisms within compositionally controlled aerosol droplets within specific atmospheric conditions. It is envisaged that within the next 10 years multidisciplinary approaches combining existing and novel techniques in atmospheric chemistry, aerobiology and molecular biology will converge and begin to dissect and empirically understand the mechanisms of microorganisms survival and decay in the aerosol state and the effect on infectivity and disease transmission.
